# Effects and Mechanisms of Five Psoralea Prenylflavonoids on Aging-Related Diseases

**DOI:** 10.1155/2020/2128513

**Published:** 2020-06-17

**Authors:** Yi-Ting Zhou, Lin Zhu, Yunyun Yuan, Shuang Ling, Jin-Wen Xu

**Affiliations:** ^1^Institute of Interdisciplinary Medical Science, Shanghai University of Traditional Chinese Medicine, Shanghai 201203, China; ^2^Clinical Medicine of Integrated Chinese and Western Medicine in Shanghai University of Traditional Chinese Medicine, Shanghai 201203, China

## Abstract

During the aging process, senescent cells gradually accumulate in the organs; they secrete proinflammatory cytokines and other factors, collectively known as the senescence-associated secretory phenotype (SASP). SASP secretions contribute to “inflammaging,” which is a state of chronic, systemic, sterility, low-grade inflammatory microenvironment and a key risk factor in the development of aging-related diseases. Fructus psoraleae is a traditional Chinese medical herb best known for delaying aging and treating osteoporosis. Prenylflavonoids from fructus psoraleae are the main bioactive compounds responsible for its pharmacological applications, such as beaching, bavachinin, bavachalcone, isobavachalcone, and neobavaisoflavone. In previous decades, there have been some promising studies on the pharmacology of fructus psoraleae. Here, we focus on the anti-inflammatory and antiaging diseases of five psoralea prenylflavonoids, such as cardiovascular protection, diabetes and obesity intervention, neuroprotection, and osteoporosis, and discuss the mechanism of these active ingredients for better understanding the material basis and drug application of fructus psoraleae in Chinese medicine.

## 1. Introduction

**Figure 1:**
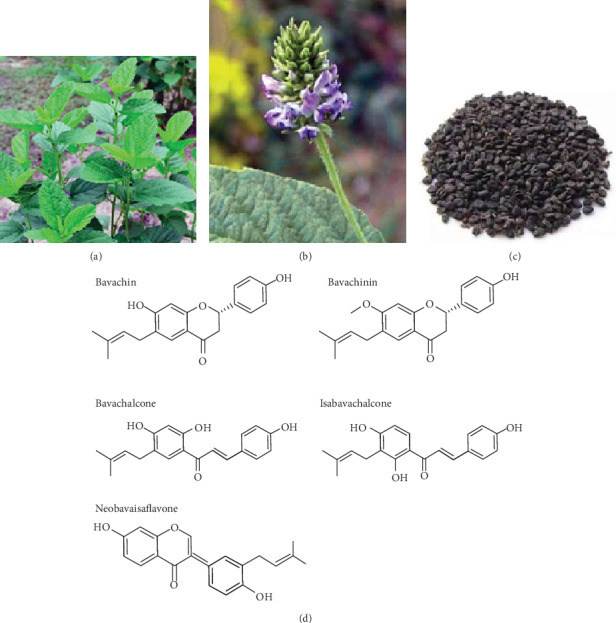
Fructus psoraleae and prenylflavonoid structures: (a) psoralea plants, (b) psoralea flowers, (c) Chinese herbal product of fructus psoraleae, and (d) prenylflavonoid structures.

Fructus psoraleae (补骨脂), a traditional Chinese medical herb, is the dried and mature fruit of *Psoralea corylifolia* Linn., which is an annual herb of Leguminosae (Figures 1(a)–1(c)). After drying, the psoraleae fruits are mixed with salt water and gently stir-baked until the fruits are slightly swollen. Then, the salt-processed fructus psoraleae could be used as a traditional Chinese medicine in the clinic. Fructus psoraleae is considered to have the efficacy of “relieving chronic morning diarrhea, warming and invigorating the Kidney-Yang, gathering the spirit, and enriching the bone marrow” in the *Compendium of Materia Medica*. According to the traditional Chinese medicine theories, fructus psoraleae has the function of delaying aging. The chemical constituents of fructus psoraleae mainly include coumarin, terpenoid phenols, and prenylflavonoids, which are the therapeutic material basis of fructus psoraleae. The structure of prenylflavonoids is characterized by the presence of isopentenyl side chains on the flavonoid skeletons. The structural types of isopentenyl are mainly 5 kinds of side chains: isopentenyl, hydroxyisopentenyl, pyran ring isopentenyl, lavender, and hydroxylavender. Most of psoralea prenylflavonoids are contained in ethyl acetate extract active fractions from *Psoralea corylifolia* [1, 2]. A recent study has shown that all the tested compounds in *Psoralea corylifolia* were absorbed into the blood plasma of rats rapidly, almost evenly distributed in the brain parenchyma, and the overall trend in the brain was basically similar to the results of plasma concentration time. Moreover, in terms of the ratio of total brain to plasma, isoprene flavonoids were easier to enter the brain than coumarin [3]. Another pharmacokinetic report also showed that rats taking oral administration and intraperitoneal injection with 20 mg/kg bavachalcone were detected with maximum plasma concentrations of 165 and 740 *μ*g/L, with a drug half-life of 1 and 1.25 h, respectively [4]. Fructus psoraleae has certain hepatotoxicity mainly caused by its constituents, such as bakuchiol, psoralen, and isopsoralen [5, 6]. Some psoralea prenylflavonoids exhibited inhibitory effects on microsomal enzymes in *in vitro* experiments. For example, acyl CoA:cholesterol acyltransferase (ACAT), an enzyme that catalyzes the esterification of cholesterol in the intestine and the production of lipoproteins in the liver, was inhibited by bavachin and isobavachalcone [7]. The following are some similar reports: (1) neobavaisoflavone, isobavachalcone, bavachinin, corylifol A, and bakuchiol blocked human carboxylesterase 2 [8]; (2) bavachalcone, bavachin, and corylifol A strongly and neobavaisoflavone, isobavachalcone, and bavachinin moderately inhibiting UGT1A1 [9, 10]; and (3) psoralidin, isobavachalcone, and neobavaisoflavone inhibiting CYP2E1 mRNA expression. Meanwhile, isobavachalcone showed a weak competitive inhibition on CYP3A4, whereas psoralidin and neobavaisoflavone exhibited significant induction of CYP3A4 mRNA expression [11].

With the development of aging, senescent cells gradually accumulate in tissues, which produce proinflammatory factors and form a low-degree of sterility and potential systemic inflammation to result in aging-related diseases. This negatively affected microenvironment is called “inflammaging,” which is the key factor connecting cell aging and the development of aging-related diseases. Modern pharmacological studies showed that bavachin, bavachinin, bavachalcone, isobavachalcone, and neobavaisoflavone (Figure 1(d)) had various pharmacological actions and could improve aging-related diseases. In this paper, we will review the effects and mechanisms of these five psoralea prenylflavonoids on ameliorating aging-related diseases, such as aging-induced chronic low-grade inflammation, cardiovascular dysfunction, diabetes, and obesity, and improving neurodegenerative diseases and osteoporosis.

## 2. Anti-Inflammatory Action

The accumulation of senescent cells with age causes a series of pathological manifestations. Nonproliferating cells occupy key cell niches and produce proinflammatory factors, which form a negatively affected microenvironment and present a senescence-associated secretory phenotype (SASP). SASP contributes to a state of sterile, systemic, low-grade inflammation, also known as “inflammaging,” which precedes many age-related diseases [12]. Considering the antiaging activity, a variety of synthetic and natural compounds has got keen interest in senolytic discovery (drugs selectively eliminating senescent cells) [13]. Fructus psoraleae is a traditional antiaging Chinese medical herb, and its resistance to “inflammaging” has become one of research concerns in antiaging field.

Psoraleae prenylflavonoids have been shown to suppress Toll-like receptor- (TLR-) or IL-1*β*-induced inflammation response. Multiple reports indicated that isobavachalcone, bavachinin, and bavachin inhibit the expression of iNOS, COX-2, and mPGES-1 and the production of nitric oxide (NO) and prostaglandin E_2_ (PGE_2_) in microglia, macrophages, and chondrocytes [14–18]. Bavachin also inhibited I*κ*B*α* degradation and increased the nuclear translocation of p65 and p50 proteins in the inflammatory chondrocytes and endothelial cells [19, 20]. In addition, bavachin significantly inhibited the activity and expression of matrix metalloproteinases (MMPs) and a disintegrin and metalloproteinase with thrombospondin motif (ADAMTS), while upregulating tissue inhibitors of metalloproteinases (TIMPs) in mouse chondrocytes [17]. These findings showed that bavachin has an anti-inflammatory effect on chondrocytes and could be used to treat articular cartilage degeneration [17, 19]. Furthermore, bavachin has shown to attenuate LPS-induced inflammation and inhibit macrophage NLRP3 inflammasome activation. Bavachin suppressed caspase-1 activation, IL-1*β* secretion, and inflammasome complex formation [18]. Isobavachalcone can significantly downregulate the levels of intercellular adhesion molecule-1 (ICAM-1) and interferon-*β* (IFN-*β*) and inhibit leukocyte adhesion to brain endothelial cells through inhibiting the TLR4/MyD88 signaling pathway [20]. Studies have determined that neobavaisoflavone significantly inhibits the production of reactive oxygen species, reactive nitrogen species, and cytokines in LPS+IFN-*γ*- or PMA-stimulated RAW264.7 macrophages, such as IL-1*β*, IL-6, and tumor necrosis factor- (TNF-) *α* [21]. A previous study has shown that isobavachalcone, bavachin, bavachinin, and neobavaisoflavone also have inhibitory effects on IL-6-induced STAT3 promoter activity and phosphorylation in Hep3B cells [22].

**Figure 2:**
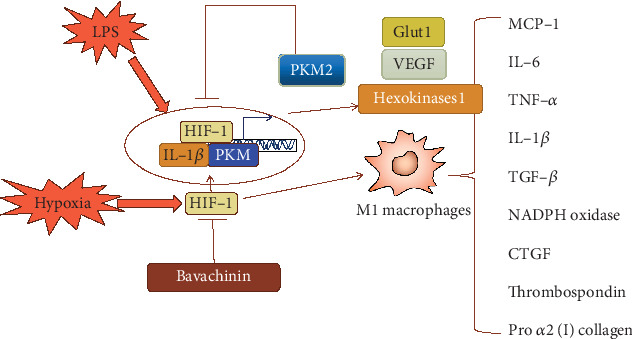
The schematic diagram of hypoxia/HIF-1*α* signal transduction pathways inhibited by bavachinin in the anti-inflammation.

Bavachinin attenuated HIF-1*α* activity under hypoxia in a concentration-dependent manner and reduced HIF-1-regulated transcription of genes related to energy metabolism, such as glucose transporter type 1 (Glut1) and hexokinase 2 [23]. HIF-1*α* is the main oxygen sensor within cells and is essential to the regulation of cell responses to varying oxygen levels. The transcription factor HIF-1*α* forms a complex relationship with inflammation and mitochondrial metabolism [24–27]. Recent studies have elucidated a new mechanism for mitochondrial metabolism to promote IL-1*β* expression and activity via HIF-1*α* regulation [25, 28]. Activated PKM2 inhibited the LPS-induced expression of HIF-1*α* and IL-1*β*, as well as a series of other HIF-1*α*-dependent genes. PKM2 induced by LPS forms a complex with HIF-1*α* and directly binds to the IL-1*β* promoter. Researchers also observed that activated PKM2 inhibited LPS-induced glycolytic reprogramming and succinate production [28]. LPS-induced succinate stabilizes HIF-1*α* which leads to activation of the proinflammatory cytokine IL-1*β* [25]. In obesity-related heart failure with preserved ejection fraction (HFpEF), HIF-1*α* is responsible for recruiting M1 macrophages that mediate obesity-associated inflammation. M1 macrophages produce proinflammatory cytokines IL-6, monocyte chemoattractant protein-1 (MCP-1), TNF-*α*, and IL-1*β* and increase the expression of thrombospondin, pro *α*2 (I) collagen, transforming growth factor (TGF)-*β*, nicotinamide adenine dinucleotide phosphate (NADPH) oxidase, and connective tissue growth factor (CTGF) [29]. Therefore, the inhibition of HIF-1*α* activity by bavachinin antagonized the inflammatory response under ischemia and hypoxia (Figure 2).

**Figure 3:**
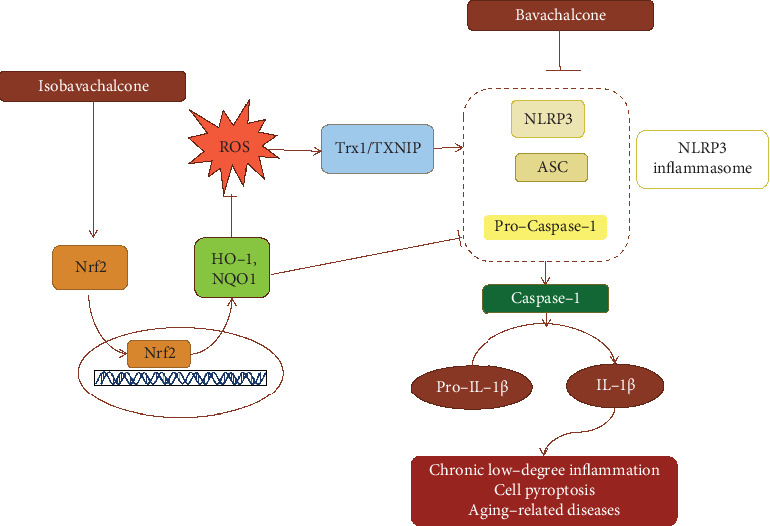
The schematic diagram of the Nrf2 signal transduction pathway activated by isobavachalcone and of NLRP3 inflammasome inhibited by bavachalcone in the anti-inflammation.

Prenylflavonoids also induced the expression and activity of transcription factors. For example, isobavachalcone antagonized lung injury and myotube atrophy by increasing the activity and expression of nuclear factor erythroid 2-related factor 2 (Nrf2) [30, 31]. Nrf2 is highly sensitive to oxidative stress, which is closely related to inflammatory response [32] and aging-related diseases, cardiovascular diseases, Alzheimer's and Parkinson's diseases, and rheumatic diseases [33–38]. Researchers demonstrated that Nrf2 inhibition of ROS-induced NLRP3 priming required the participation of NAD(P)H quinone dehydrogenase 1 (NQO1) [39]. Furthermore, the inhibition of Nrf2 also regulated the NLPR3 inflammasome formulation, assembly-mediated cleaved caspase-1, and IL-1*β* secretion through the Trx1/TXNIP complex [40]. In addition, Nrf2 binding to antioxidant responsive element (ARE) site can trigger the transcription of more than 200 endogenous protective genes, such as antioxidant, anti-inflammatory, and antiapoptotic genes [33, 41]. Therefore, the moderate expression and balance of Nrf2 promoted by prenylflavonoids are beneficial to the regulation of antioxidant, anti-inflammatory, and mitochondrial homeostasis (Figure 3).

Our latest study has shown that 4'-O-methybavachalcone significantly reduced cerebral infarction and edema, improved neurobehavioral indexes, inhibited the production of IL-1*β*, TNF-*α*, and IL-6 in ischemic brain, and reduced the level of NLRP3, cleaved caspase1, and GSDMD-N (gasdermin D N-terminal domain) in the mouse stroke model and in oxygen glucose deprivation- (OGD-) RAW264.7 cells [42]. Recently, gasdermin D and NLRP3 have been identified as programmed death patterns of stroke cell inflammation and pyrolysis [43]. Moreover, a specific pyroptosis inhibitor Vx765 has a potential therapeutic value for cerebral ischemia by inhibiting the canonical inflammasome pathway of pyroptosis [44]. In brief, bavachalcone has the ability to inhibit the inflammatome NLRP3 activity and cell pyrolysis (Figure 3).

It has been reported that the bavachin activated gene expression of peroxisome proliferator-activated receptor gamma (PPAR*γ*) [45]. As we all know, PPAR*γ* can transform proinflammatory cytokines produced by neutrophils, platelets, and macrophages into anti-inflammatory mediators. PPAR*γ* and its ligands further regulate platelet and neutrophil function, reduce trafficking, promote neutrophil apoptosis, and prevent platelet-leukocyte interactions. PPAR*γ* alters macrophage transport, increases efferocytosis and phagocytosis, and promotes macrophages to switch to M2-type polarity [46]. What is more, endothelial PPAR*γ* is an indispensable factor to prevent endothelial dysfunction with aging [47], and interference with PPAR*γ* function can accelerate vascular aging and atherosclerosis [48, 49]. Conversely, cell senescence may also alter the PPAR*γ*-dependent fatty acid handling in human microvascular endothelial cells and contribute to inflammation [50].

In the following summary (Table 1), the cell or animal models, inflammation induction methods, active recipients, experimental doses, and pharmacological effects in more detail are provided. In general, prenylflavonoids inhibited JNK1/2, ERK1/2, NF-*κ*B, STAT3, inflammatome NLRP3 activity, iNOS and mPGES-1 expression, secretion of many cytokines, and cell pyroptosis. These findings indicate that psoralea prenylflavonoids have the potential effects on improving “inflammaging” environment and aging-related diseases.

## 3. Cardiovascular Function Regulation

The age-related status changes lead to low-degree inflammation and increase of oxidative stress in the cardiovascular system. The phenotypes of these changes include rigidity of elastic arteries, endothelial dysfunction, hypoperfusion cerebral ischemia, stroke, and cardiac diastolic dysfunction. This involves the Klotho longevity gene, the AMPK energy-sensitive pathway, and the activity and expression of eNOS, MnSOD, and PGC-1*α* genes.

The cardiovascular protective effects of prenylflavonoid derivatives from fructus psoraleae have been reported (Table 2). Kassahun et al. [51] reported that isobavachalcone inhibited hTRPC3 current, which significantly relaxes the contraction of the endothelium-intact aortic rings induced by phenylephrine. In addition, a previous study has shown that isobavachalcone inhibits platelet aggregation induced by arachidonic acid, collagen, and platelet activating factor in rabbits [52]. As we all know, platelet aggregation is closely related to myocardial infarction and stroke [53, 54].

**Figure 4:**
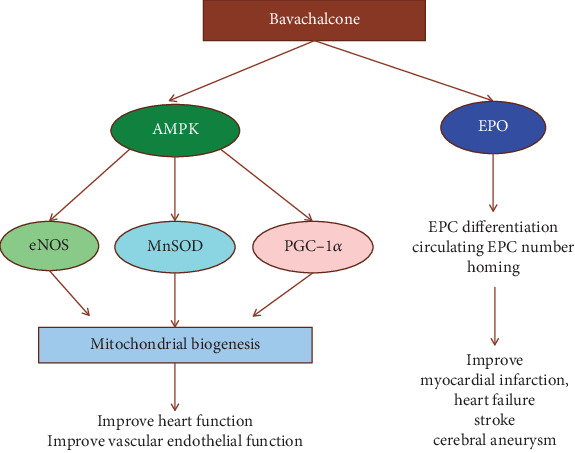
The schematic diagram of bavachalcone improving cardiovascular function by activating AMPK and promoting EPO expression.

Acyl-CoA:cholesterol acyltransferase (ACAT), a key enzyme regulating cholesterol esterification, induced the accumulation of cholesterol esters in the endoplasmic reticulum of macrophages and smooth muscle cells and promoted the formation of foam cells [55, 56]. However, the enzyme was inhibited by isobavachalcone and bavachin in an ACAT noncompetitive inhibition type [7]. In *in vitro* studies performed by our team in human endothelial cells, bavachalcone suppressed cell senescence by decreasing the activity of a senescence-associated *β*-galactosidase (a senescent markers) and downregulating mRNA expression of p16 (ink4a) (a marker of replicative senescence) and IL-1*α* (a proinflammatory cytokine of SASP) [57]. Bavachalcone also activated retinoid-related orphan receptor alpha 1 (ROR*α*1) reporter gene activity and induced ROR*α*1 mRNA expression in a dose-dependent manner, resulting in the enhancement of the amplitude of circadian rhythm of Bmal1 mRNA expression after serum shock [57]. Another study has shown that bavachalcone protected endothelial cells by increasing the activity of adenosine 5′-monophosphate-activated protein kinase (AMPK) and the expression of manganese-superoxide dismutase (MnSOD) and PGC-1*α*, inhibiting mitochondrial oxidative stress and improving mitochondrial biogenesis [58]. We also have found that bavachalcone promoted endothelial progenitor cell (EPC) differentiation and neovascularization through the AMPK pathway. Oral administration of bavachalcone for 14 days stimulated neovascularization and reestablishment of microcirculation in a rat ischemic hind limb model. The number of EPCs and the level of erythropoietin (EPO) in the circulation were increased in rats with bavachalcone treatment. Furthermore, bavachalcone enhanced the activity of ROR*α*1 and EPO reporter genes [59]. Bahlmann et al. [60] reported that the treatment of recombinant human EPO caused a significant mobilization of circulating CD34(+)/CD45(+) EPC in the peripheral blood and increased the number of functionally active EPCs in patients and healthy subjects. Previous clinical studies and animal experiments also have shown that EPO therapy increased the number of circulating EPCs and promoted homing and improved acute myocardial infarction, heart failure, stroke, and cerebral aneurysm [61–64]. Our multiple results indicate (Figure 4) that AMPK-mediated cardiovascular protection of bavachalcone can include regulating mitochondrial biogenesis [65], delaying cell senescence [66], activating eNOS, MnSOD and PGC-1*α* activity and expression [67, 68], and promoting EPC differentiation [69], which presents consistency with the results of other research groups.

## 4. Diabetes and Obese Intervention

Aging, diabetes, and obesity had a synergistic effect on inflammation and oxidative stress among the elderly, which in turn increased the risk of diabetes and obesity. Early interventions to reduce inflammation, oxidative stress, and insulin resistance may improve age-related diseases [70–73]. Fructus psoraleae, as an option for seeking treatment and prevention of diabetes and obesity, has the potential to improve the active life expectancy of the elderly.

A recent report revealed that psoralea prenylflavonoid extract (PFE) significantly reduced body weight and fat mass in a dose-dependent manner, increased energy expenditure, improved insulin sensitivity, and prevented hepatic steatosis by increasing lipid oxidation and secretion in high-fat diet-fed mice. The study also found that PFE induced clear browning in subcutaneous white adipose tissue, increased brown adipose tissue activity, and thermogenic genes [74]. Another research group also reported that the extract of Psoralea corylifolia seeds attenuated methylglyoxal-induced insulin resistance and significantly increased the phosphorylation of Akt, IRS-1/2, and glucose uptake [75]. Moreover, this extract reduced nonalcoholic fatty liver disease, the expression of lipogenic, and inflammatory genes, such as TNF-*α*, IL-1*β*, and iNOS in high-fat diet-induced obese mice [76]. In addition, Lee et al. [45] showed that bavachin (2 *μ*M) increased the expression and secretion of adiponectin and enhanced glucose uptake via GLUT4 translocation by activating the protein kinase B (Akt) and AMPK pathway in the presence or absence of insulin. Targeting the AMPK signaling pathway is one of the target pathways to control diabetes. Some studies have shown that diabetic patients have impaired AMPK activity, and metformin, which is used to treat diabetes, works by regulating AMPK. Natural medicines of plant origin showed great potential in regulating and activating the AMPK pathway and can be used as drug candidates for the treatment of diabetes and its complications [77, 78].

**Figure 5:**
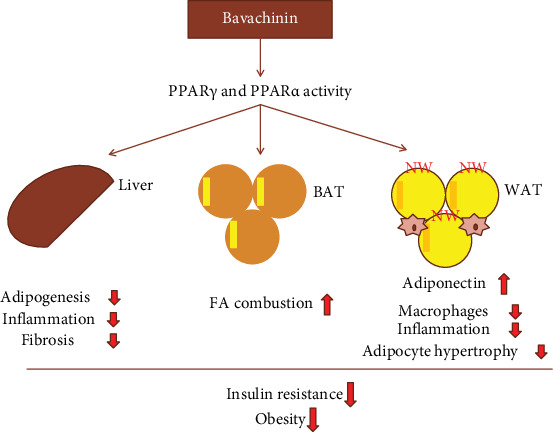
The schematic diagram of bavachinin activating PPARs to improve insulin resistance and obesity.

Moreover, bavachin promoted lipid accumulation in 3T3-L1 cells at differentiation day 8 by activating gene expression of peroxisome proliferator-activated receptor gamma (PPAR*γ*) and CCAAT/enhancer binding protein alpha (C/EBP*α*) [45], suggesting that bavachin might have therapeutic potential for type 2 diabetes. Like bavachin, bavachinin is a PPAR*γ* agonist. Isolated bavachinin is a mixture of *S* and *R* configurations. A time-resolved fluorescence resonance energy transfer-based competitive binding assay showed that (*S*)- and (*R*)-bavachinin had similar PPAR*γ* agonist activities with IC_50_ 616.7 nM and 471.2 nM, respectively [79]. Subsequent *in vivo* studies have shown that bavachinin exhibited blood glucose-lowering properties without causing weight gain and liver toxicity. Importantly, bavachinin had synergistic effects with the synthetic PPAR*γ* agonist thiazolidinediones and PPAR*α* agonist fibrates, which induced PPAR transcriptional activity, as well as lowered glucose and triacylglycerol levels in db/db mice [80]. Further research found that bavachinin bound not only to the canonical site of PPAR agonists but also to a novel alternative binding site. Moreover, rosiglitazone pretreatment prevented bavachinin from binding to the canonical site instead of a novel alternative binding site [80]. The sketch of bavachinin activating PPARs to improve insulin resistance and obesity is shown in Figure 5.

Isobavachalcone caused 3T3-L1 cell cycle arrest at G0/G1 and dose-dependently inhibited the protein expression levels of cyclin D1, cdk4, and cd6 within 48 h after adipocyte differentiation medium MDI stimulation. Moreover, while isobavachalcone inhibited adipocyte fat accumulation, it also reduced intrahepatic fat deposition and hepatic steatosis in zebrafish fed high fat cholesterol diets [81]. Table 2 summarizes the experimental data related to diabetes and obesity.

## 5. Neuroprotection

Aging is the most important independent risk factor for neurodegenerative diseases. Millions of people suffer from Alzheimer's disease (AD), Parkinson's disease (PD), vascular dementia (VD), and ischemic stroke around the world. Fructus psoraleae is a promising antiaging Chinese herb which is introduced for the therapy of neurodegenerative diseases. Several studies have demonstrated that *Psoralea corylifolia* seed extracts can exert neuroprotection *in vivo* and *in vitro*. Alzheimer's disease (AD) is an age-related neurodegenerative disease. Evidence showed that four components of *Psoralea corylifolia*, named bavachin, bavachinin, bavachalcone, and isobavachalcone, differentially inhibited neuroinflammation, oxidative damage, and key AD-related protein targets, such as amyloid *β*-peptide 42, *β*-secretase, glycogen synthase kinase 3*β*, and acetylcholinesterase *in vivo* [82]. The researchers further revealed that isobavachalcone and bavachinin regulate the aggregation of a*β*42 through different mechanisms. Isobavachalcone significantly inhibited both oligomerization and fibrillization of A*β*42, whereas bavachinin inhibited fibrillization and led to off-pathway aggregation. Both of the compounds attenuated A*β*42-induced toxicity in an SH-SY5Y cell model [83]. Meanwhile, isobavachalcone can also effectively relieve Parkinson's disease (PD) induced by 1-methyl-4-phenyl-1,2,3,6-tetrahydropyridine (MPTP), prolong the residence time of mice on Rota-rod, and alleviate the neuronal necrosis [84]. Isobavachalcone also inhibited the activation of microglia and decreased the expression of IL-6 and IL-1*β* in the brain of PD mice [80]. *In vitro*, isobavachalcone significantly suppressed BV-2 microglia activation and the NO production induced by LPS [84], while neobavaisoflavone had a strong inhibitory activity on H_2_O_2_-induced cell death in HT22 hippocampal cells [85]. Monoamine oxidase B (MAO-B) is an enzyme that breaks down dopamine in the brain. MAO-B inhibitors block the action of the enzyme and therefore improve the symptoms in early PD. A molecular docking test found that the C7 methoxy group of bavachinin has a high affinity for MAO-B, suggesting that bavachinin might be a selective and competitive human MAO-B inhibitor and could be used in the PD treatment [86]. The experimental details are summarized in Table 2.

Recently, bavachinin was identified as a novel agonist that activates all three PPAR isoforms, so-called pan-PPAR agonist [80], which indicated its potentially powerful application of prenylflavonoids in neuroprotection. PPAR*γ* agonists have been demonstrated as neuroprotective agents. Pioglitazone and 15d-prostaglandin J2 significantly reduced the infarct volume, attenuated the aggregation of parenchymal neutrophils, decreased the release of TNF-*α*, IL-1*β*, and IL-6, and improved neurological function in the cerebral ischemia injury mice [87, 88]; reduced *β*-amyloid levels effectively reversed cerebrovascular damage, improved energy metabolism and antioxidant capacity of model mice's cortex, and inhibited *β*-amyloid-stimulated COX-2, TNF-*α*, and IL-6 expression in Alzheimer's disease models [89–91]. Pioglitazone and MDG548 also increased the phagocytosis of microglia on necrotic cells, reduced the death of dopaminergic neurons and the production of cellular reactive oxygen species, upregulated the expression of MRC1, CD68, and IL-10, elevated the activity of glutathione-S-transferase, and blocked the activity of monoamine oxygenase B in the Parkinson's disease MPTP-mice [92–94].

**Figure 6:**
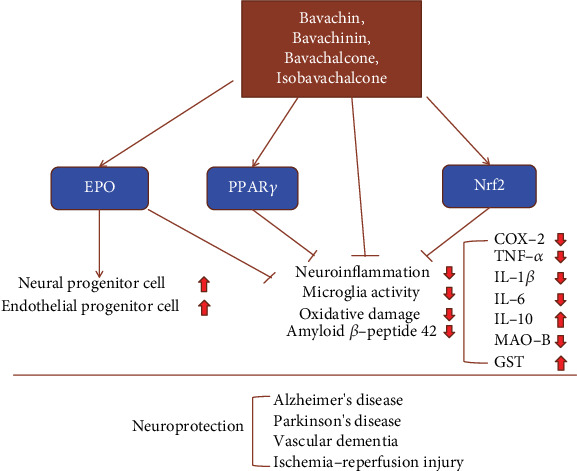
The schematic diagram of the neuroprotective effects of multiple prenylflavonoids by promoting EPO, Nrf2, PPAR*γ* activation, and direct anti-inflammatory effects.

Endogenous Nrf2 is the main regulator of the antioxidant response and an indicator of oxidative stress. Numerous research data indicated that Nrf2 mediated the neuroprotective effect of active ingredients of Chinese herbal medicine on Parkinson's disease [95, 96], Alzheimer's disease [97, 98], vascular dementia [99, 100], and cerebral ischemia-reperfusion injury [101–103]. *In vivo* and *in vitro* studies suggested that isobavachalcone may act as a potential neuroprotective agent by increasing Nrf2 activity and expression [30, 31]. The therapeutic potential of EPO in brain plays a neuroprotective role against Alzheimer's disease [104, 105], Parkinson's disease [106–108], chronic cerebral hypoperfusion-induced vascular dementia [109, 110], and cerebral ischemia-reperfusion injury [111–113]. The elevation of expression and circulating concentration of EPO improved the therapeutic effects of bavachalcone and other flavonoids of Chinese herbal medicine in brain diseases [59, 114–116]. The neuroprotective effects of psoralea prenylflavonoids are summarized in Figure 6.

## 6. Improve Osteoporosis

Senile osteoporosis is caused by aging and affects both men and women. The evidences showed that (1) senescence of bone marrow stromal cells (BMSCs) was more likely to differentiate into adipocytes rather than osteoblasts [117, 118]; (2) age-dependent bone loss is associated with increased bone cell DNA damage, cell aging, SASP, and elevated RANKL levels [119–121]; and (3) excessive osteoclast activity induced by aging is closely related to inflammation, which induced osteoporosis, while anti-inflammatory drugs inhibit osteoclast formation and bone resorption [122–124].

**Figure 7:**
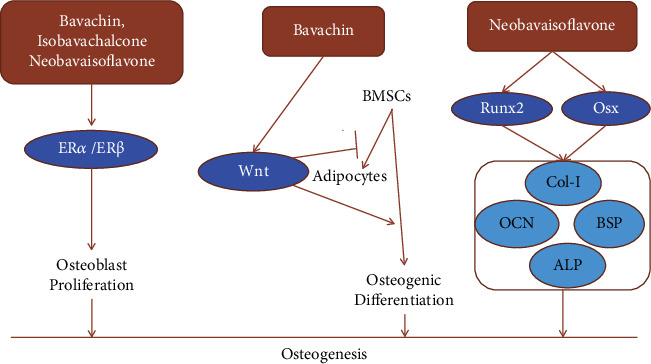
The schematic diagram of multiple prenylflavonoids promoting the differentiation of osteoblasts via regulating ER*α*/*β*, Wnt, Runx2, and Osx.

In China, fructus psoraleae is often used to treat fractures, osteomalacia, and osteoporosis. A phytoestrogen effect was considered to play an important role in studies on the material basis of fructus psoraleae and its pharmacological action (Table 2). Xin and colleagues [125] found that bavachin, isobavachalcone, and neobavaisoflavone activate estrogen receptor ER*α* and ER*β*, while pure estrogen antagonists ICI-182,780 completely block the activation of estrogen receptors. Bavachin, isobavachalcone, and neobavaisoflavone also significantly promoted osteoblast proliferation [126]. In a model of bone loss caused by ovariectomy, *in vivo* experiments showed that bavachin prevents bone loss by up-regulating the Wnt signaling pathway [127]. Wnt/*β*-catenin signaling pathway regulated cell fate and promoted BMSCs transfer to osteoblasts rather than adipocytes [128, 129]. Neobavaisoflavone stimulated osteogenesis of MC3T3-E1 osteoblasts in a dose-dependent manner, which is manifested by notable enhancement of ALP activity, significantly increased bone-specific matrix proteins including type I collagen (Col-I), osteocalcin (OCN), and bone sialoprotein (BSP) and formation of bone nodules. Neobavaisoflavone also upregulated the mRNA expression of transcription factors Runx2 and Osterix (Osx) through a p38-dependent signaling pathway then exerted osteogenic activity [130]. Runx2, ALP, and OCN are integral factors for the control of osteogenesis. Many research reported that Runx2 can promote osteogenic differentiation and inhibit fat differentiation [131, 132]. As an osteoblast-specific transcription factor, Osx is also required for osteoblast differentiation. Osx showed a transcriptional regulatory mechanism for BSP gene expression, which is related to osteoblast differentiation and the onset of mineralization [133]. Interestingly, aiming at 162 potential bone-promoting target proteins, Ge et al. [134] revealed that 8 ingredients of 23 known psoralen components, such as bavachalcone, bavachinin, and neobavaisoflavone, may be the main activities of psoralea-promoting bone formation in MC3T3-E1 cells. In summary, psoralea prenylflavonoids promoted bone formation in many ways (Figure 7).

**Figure 8:**
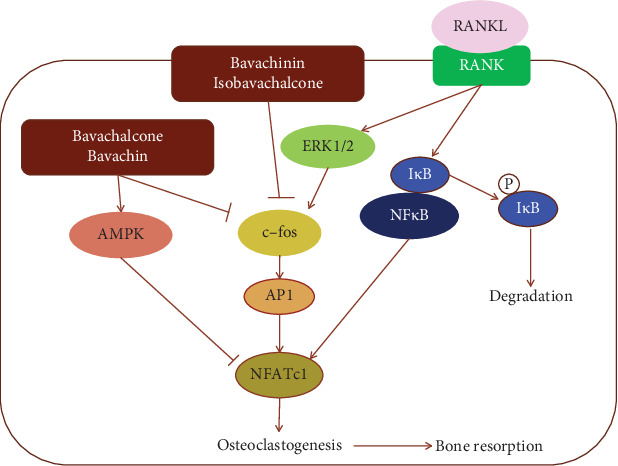
The schematic diagram of multiple prenylflavonoids inhibiting osteoclast differentiation and bone resorption induced by RANKL.

Contrary to osteoblasts, osteoclast is a kind of cell that has a special effect on bone absorption. Disorder of bone absorption is the main culprit of many bone diseases such as osteoporosis. Bavachalcone inhibited the differentiation of precursor cells into osteoclasts by interfering with the extracellular regulated protein kinases (ERK) and Akt pathways and suppressed the expression of c-Fos and NFATc1 via receptor activator of nuclear factor *κ*B ligand (RANKL) [135]. NFATc1 induced by the cytokine RANKL is a major regulator of the osteoclast transcriptome. NFATc1 promoted the expression of many genes, such as Itgb3, Oscar, Acp5, and Ctsk promoters, which were required for osteoclastogenesis and bone resorption [136]. NFATc1 protein expression and transcriptional activity also played important roles in inflammatory osteoclastogenesis and pathological bone loss [137, 138]. An earlier study reported that bavachalcone can intervene with osteoclastogenesis by inhibiting the induction of c-Fos and NFATc1 [135]. AMPK, a negative regulator of NFATc1 activation, antagonized osteoclastogenesis [139, 140]. Estrogen and phytoestrogens also negatively regulated the RANKL-induced osteoclastic differentiation [141–143]. Many studies revealed that psoralea prenylflavonoids activate the AMPK pathway and present estrogen-like effects, which explained their inhibitory mechanism on osteoclastogenesis (Figure 8).

In conclusion, this article summarizes the numerous pharmacological effects of psoralea prenylflavonoids on aging-related diseases, including anti-inflammatory action, cardiovascular protection, diabetes and obese intervention, neurodegenerative diseases improvement, and osteoporosis alleviation. This article also discusses the regulation of these active ingredients on the key pathways, such as AMPK, EPO, HIF-1*α*, NFATc1, NF-*κ*B, Nrf2, Osx, PPAR*γ*, Runx2, and Wnt/*β*-catenin, as well as inflammatome NLRP3. However, there is insufficient evidence to prove that these five psoralea prenylflavonoids can be used as senolytics (eliminating senescent cells) or as senomorphics (modifying SASP components) drugs and can only be considered to prevent certain aging-related signaling pathways by entering cells. The summary and discussion of the pharmacological effects and regulatory mechanisms of psoralea prenylflavonoids will help to improve the recognition of the material basis of the pharmaceutical function of fructus psoraleae and will have a positive significance for the interpretation of its pharmacological effect.

## Figures and Tables

**Table 1:** Summary of anti-inflammation experiments of five psoralea prenylflavonoids.

Cells or animals	Model method	Active ingredient	Dosage	Pharmacological effect	Reference number
BV-2 microglia	LPS	Isobavachalcone	2, 5, 10 *μ*mol/L	(i) Concentration-dependent inhibitory effects on NO and PGE_2_ production with an IC_50_ for inhibiting NO production: 1.6 ± 0.11 *μ*mol/L (ii) Blocked the I-*κ*B*α* degradation and downregulated NF-*κ*B activity (iii) Inhibited the iNOS and COX-2 mRNA and protein expression	[14]

RAW264.7 murine monocytic cells	MALP-2; LPS Poly[I:C]	Isobavachalcone	20, 50, 100 *μ*mol/L	(i) Inhibited iNOS expression in luciferase reporter and protein level induced by TLRs agonists and inhibited nitrite production	[16]

Microglial cells	LPS	Bavachinin Isobavachalcone Neobavaisoflavone	1, 3, 10, 30, 100 *μ*mol/L	(i) Concentration-dependent inhibitory effects on NO production with an IC_50_: 26 *μ*mol/L (bavachinin), 17 *μ*mol/L (isobavachalcone), 29 *μ*mol/L (neobavaisoflavone)	[15]

bEnd.3 murine brain endothelial cells	MALP-2; LPS Poly[I:C]	Isobavachalcone	0.1, 1, 5 *μ*mol/L	(i) Downregulated ICAM-1 mRNA and protein expression (ii) Suppressed NF-*κ*B activity (iii) Dose-dependently attenuated the adhesion of monocytes to endothelial cells activated by LPS	[20]

Chondrocytes	5 ng/ml IL-1*β*	Bavachin	1, 2.5, 5, 10, 20 *μ*mol/L	(i) Decreased I*κ*B*α* kinase and NF-*κ*B DNA-binding activity (ii) Inhibited IL-1*β*-induced RANTES, MCP-2, MIP1-*α*, and MIP1-*β* chemokine production (iii) Reduced migration of THP-1 monocytic cells	[19]

Primary rat chondrocytes	Non	Bavachin	5, 10 *μ*mol/L	(i) Upregulated aggrecan and collagen type II expression in a dose-dependent manner (ii) Inhibited MMP-1/3/13, and ADMATS-4/5 expression, and upregulated TIMP-1/2/3/4 expression (iii) Inhibited iNOS and COX-2 expression and downregulated NO and PGE_2_ in a dose-dependent manner	[17]

Murine J774A.1 cells and murine peritoneal macrophages	LPS	Bavachin	10, 20, 30, 40 *μ*mol/L	(i) Inhibited iNOS and mPGES-1 expression and downregulated NO and PGE_2_ in a dose-dependent manner (ii) Inhibited phosphorylation of JNK 1/2 and ERK 1/2, and downregulated NF-*κ*B activity (iii) Suppressed production of IL-*β* and the expression of NLRP3 inflammasome complex (iv) Inhibited production of NO, IL-6 and IL-12p40 in LPS-stimulated murine peritoneal macrophages	[18]

RAW264.7 macrophages	LPS + IFN-*γ*	Neobavaisoflavone	2.5, 5, 10, 25, 50 *μ*mol/L	(i) Inhibited the production of ROS, RNS, and cytokines: IL-1*β*, IL-6, IL-12p40, IL-12p70, and TNF-*α* with ED_50_: 25.00 *μ*mol/L (NO); 23.11 *μ*mol/L (IL-1*β*); 5.03 *μ*mol/L (IL-6); 5.23 *μ*mol/L (IL-12p40); 5.26 *μ*mol/L (IL-12p70); 18.80 *μ*mol/L (TNF-*α*)	[21]

Hep3B cells	IL-6	Bavachin Bavachinin Isobavachalcone Neobavaisoflavone	10, 30, 60 *μ*mol/L	(i) Inhibited STAT3 phosphorylation with an IC_50_ for STAT3-dependent promoter activity: 4.89 ± 0.05 *μ*mol/L (bavachin), 3.02 ± 0.53 *μ*mol/L (bavachinin), 2.45 ± 0.13 *μ*mol/L (isobavachalcone), 2.77 ± 0.02 *μ*mol/L (neobavaisoflavone)	[22]
Male ICR mice	Ischemic stroke	4'-O-methybavachalcone	5, 10, 20 mg/kg, oral gavage after animal modeling, daily	(i) Improved the area of cerebral infarction, brain edema, and neurobehavioral indexes 48 hours after MCAO/R (ii) Lowered active-PARP and cleaved-caspase-3 levels (iii) Inhibited IL-1*β*, TNF-*α*, and IL-6 production in ischemic cerebral homogenate (iv) Reduced NLRP3/GSDMD-mediated pyroptosis of brain tissue and cells	[43]

**Table 2:** Experimental summary of five psoralea prenylflavonoids improving aging-related diseases.

Classification	Model	Stimulation	Active ingredient	Dosage	Pharmacological effect	Reference number
Cardiovascular function regulation	Male SD rat aortic rings and HEK293 cells	Exposure to 60 *μ*mol/L KCl followed by two exposures to 1 *μ*mol/L phenylephrine to precontract rings	Isobavachalcone	50 *μ*mol/L for aortic rings and 150 *μ*mol/L for hTRPC3 test	(i) Caused the relaxation of precontracted aortic rings in the presence of endothelium (ii) Isobavachalcone-induced vasodilation was blocked by NOS inhibitor L-NAME and SGC blocker ODQ (iii) Inhibited hTRPC3 currents (27.1 ± 7.9%)	[51]
	Platelets from fresh rabbit blood	Induction with collagen (col), arachidonic acid (AA), or platelet-activating-factor (PAF)	Isobavachalcone Neobavaisoflavone	2, 5 or 80 nmol/L	(i) Concentration-dependent inhibition of platelet aggregation (ii) IC_50_ for isobavachalcone: 65.1 ± 9.1 *μ*mol/L (col), 0.5 ± 0.1 *μ*mol/L (AA), and 41.6 ± 6.2 *μ*mol/L (PAF); IC_50_ for neobavaisoflavone: 62.4 ± 10.4 *μ*mol/L (col), 7.8 ± 2.5 *μ*mol/L (AA), and 2.5 ± 0.3 *μ*mol/L (PAF)	[52]
	Rat liver microsome And HepG2 cells	Non	Bavachin Isobavachalcone	3.0, 9.3, 30, and 8, 93.0, 300 *μ*mol/L	(i) Inhibits ACAT activity (ii) IC_50_ values were 86.0 *μ*mol/L (bavachin) and 48.0 *μ*mol/L (isobavachalcone) in the ACAT assay system (iii) Isobavachalcone inhibited cholesteryl ester formation in a dose-dependent fashion with an IC_50_ value of 100.2 *μ*mol/L in HepG2 cells	[7]
	HUVEC	Induction of senescent cells by hydrogen peroxide	Bavachalcone	2.5, 5, 10, 20 *μ*mol/L	(i) Inhibited acidic beta-galactosidase activity (ii) Induced ROR*α*1 expression in ROR*α* reporter luciferase activity, mRNA, and protein levels in a dose-dependent manner and enhanced the circadian amplitude of Bmal1 mRNA expression after serum shock (iii) Suppressed senescence in human endothelial cells and mRNA expression of p16 (ink4a) (a marker of replicative senescence) and IL-1*α* (a proinflammatory cytokine of the senescence-associated secretory phenotype)	[57]
	HUVEC	Induction of senescent cells by hydrogen peroxide	Bavachalcone	1, 2, 5 *μ*mol/L	(i) Inhibited acidic beta-galactosidase activity (ii) Promoter and increased MnSOD mRNA and protein expressions (iii) Suppressed the mitochondrial superoxide production in endothelial cells (iv) Stimulated liver kinase B1 and AMPK*α* phosphorylation (v) AMPK knockdown by shRNA-AMPK reversed the effects of bavachalcone on MnSOD	[58]
	(1) HUVECs (2) EA.hy926 cells (3) Rat bone marrow mesenchymal cells (4) Male Wistar rat	Rat ischemic hindlimb model	Bavachalcone	1, 2, 5 *μ*mol/L for cell culture and 3 mg/kg daily intragastric administration for in vivo experiments	(i) Low-dose bavachalcone administered orally for 14 days stimulated the recovery of ischemic hindlimb blood flow in rat hindlimb ischemia models (ii) Increased circulating EPCs and promoted capillary angiogenesis (iii) Bavachalcone treatment of rat bone marrow cells for 24 h initiated the AMP-activated protein kinase activity (iv) Enhanced the activity of ROR*α*1 and EPO luciferase reporter gene (v) Bavachalcone treatment elevated EPO mRNA and protein expression in vitro and in vivo and the circulating EPO levels in rats (vi) Bavachalcone induced differentiation of bone marrow cells into endothelial progenitor cells (vii) Increased number of EPCs and the level of EPO in the circulation in rats	[59]
Diabetes and obese intervention	Mouse 3T3-L1 pre-adipocytes	Media containing MDI (1 *μ*g/mL isobutyl-methylxanthine, 1 *μ*M dexamethasone, and 1 *μ*g/mL insulin)	Bavachin	2 *μ*mol/L	(i) Increased the expression and secretion of adiponectin (ii) Enhanced glucose uptake via GLUT4 translocation	[45]
	9-week-old female db/db mice	High-fat diet	Bavachinin	50 and 100 mg/kg/day	(i) Bavachinin dose-dependently induced the transcriptional activities of the mouse ligand-binding domain of PPAR*γ* and PPAR*α* (ii) Ameliorates diabetes and hyperlipidaemia in db/db and in diet-induced obese mice (iii) Decreased lipid accumulation in liver in db/db and in diet-induced obese mice (iv) Regulates PPAR gene expression in vitro and in vivo (v) Occupies a novel alternative binding site in addition to the canonical site of synthetic agonists of PPAR*γ*	[80]
	3T3-L1 preadipocytes and Zebrafish	Media containing MDI (1 *μ*g/mL isobutyl-methylxanthine, 1 *μ*M dexamethasone, and 1 *μ*g/mL insulin)	Isobavachalcone	2, 10, 40 *μ*mol/L	(i) Decreased protein levels of PPAR*γ* and C/EBP*α* (ii) Gene expression levels of SREBP1c, adiponectin, ACC1, and FAS (iii) Inhibited adipogenesis and prevents lipid accumulation in high cholesterol-diet Zebrafish larvae	[81]
Neuroprotection	BV-2 microglia	LPS 450 *μ*mol/L of H_2_O_2_ solution	Bavachin Bavachinin Bavachalcone Isobavachalcone	1, 10, 100 *μ*mol/L	(i) Inhibited neuroinflammation, oxidative damage, and key AD-related protein targets in BV-2 microglia (ii) Inhibited *β*-secretase (BACE-1), glycogen synthase kinase 3*β* (GSK-3*β*), and acetylcholinesterase (AChE) expression (iii) Bavachalcone and isobavachalcone exhibited strong inhibitory activities on A*β*42 aggregation	[82]
	SH-SY5Y cell Yeast two hybrid	A*β*42	Isobavachalcone (IBC) Bavachinin (BCN)	3 *μ*mol/L (IBC) and 30 *μ*mol/L (BCN)	(i) Inhibited A*β*42 aggregation (ii) Attenuated A*β*42-induced cell toxicity	[83]
	Mouse BV-2 cells Neuro-2a cells Male C57BL/6 mice	LPS Preadministration of MPTP (20 mg/kg)	Isobavachalcone	0.1, 1, 5, 10, 20 *μ*mol/L 50 mg/kg	(i) Improved the motor, balance, and coordination abilities of PD mouse (ii) Inhibited the activation of microglia and astrocytes and the necrosis of neurons injured by MPTP (iii) Decreased the levels of IL-6 and IL-1*β* in PD mouse and of TNF-*α*, IL-6, IL-1*β*, and IL-10 in BV-2 cells stimulated by LPS (iv) Inhibited the activation of p65 subunit in brain tissues the expression of p65 in BV-2 cells (v) Inhibited the levels of NO and the expression of iNOS in LPS-treated BV-2 cells	[84]
	BV-2 cells HT22 hippocampal cells	LPS (1 *μ*g/mL) 250 *μ*mol/L of H_2_O_2_	Isobavachalcone Neobavaisoflavone	5, 10, 20 *μ*mol/L	(i) Protective effects against H_2_O_2_-induced neuronal cell damage in HT22 hippocampal cells (i) Isobavachalcone had the most significant inhibitory effect on the LPS-induced NO production in dose-dependent manners in BV-2 cells	[85]
	Human hMAO-A and hMAO-B	Non	Bavachinin	10, 20, and 40 *μ*mol/L or 18.75, 75, and 300 *μ*mol/L	(i) Selectively inhibited MAO-A and MAO-B activity (ii) With IC_50_ of 8.82 *μ*mol/L (hMAO-B) and 189.28 *μ*mol/L (hMAO-A) (iii) Bavachinin C7-methoxy group had higher affinity for hMAO-B using molecular docking examination	[86]
Regulation of bone formation and absorption	HeLa cells	Non	Bavachin Isobavachalcone Neobavaisoflavone	10^−9^, 10^−8^, 10^−7^, 10^−6^ mol/L	(i) Activated transcription of ER*α* or ER*β* in HeLa cells	[126]
	Female SD rats Human osteoblasts	Ovariectomy	Bavachin	8 mg/kg/day 0.5, 5, 50 *μ*mol/L	(i) Increased the thickness and integrality of femur cortical bone (ii) Increased the mRNA level of OPG in the OVX femur (iii) Promoted the proliferation of human osteoblasts (iv) Induced primary human osteoblast differentiation by upregulated the Wnt3a/*β*-catenin signaling pathway (v) Increased ALP, Runx2, OCN, Col-I, and LRP5 expression	[127]
	MC3T3-E1cells	Non	Neobavaisoflavone	1, 5, 10, 15, 20 *μ*mol/L	(i) Induced mineralization in MC3 T3-E1 cells (ii) Upregulated the expression of Runx2 and Osx (i) Induced activation of ALP, the expression of Col-I, OCN, and BSP	[130]
	Bone marrow-derived macrophages	RANKL Vitamin D3 (10^−6^ mol/L) and PGE2 (10^−8^ 2mol/L).	Bavachalcone	0.5, 1, 2, 5 *μ*mol/L	(i) Inhibited osteoclastogenesis in coculture of whole bone marrow cells and calvarial osteoblasts and inhibited bone resorption (ii) With the IC_50_ of approximately 1.5 *μ*mol/L (iii) Suppressed the expression of c-Fos and NFATc1 by RANKL (iv) Reduced activation of MEK, ERK, and Akt by RANKL	[135]

## Abbreviations

A*β*42: A 42 amino acid proteolytic product from the amyloid precursor protein
ACAT: Acyl-CoA:cholesterol acyltransferase
AD: Alzheimer's disease
ADAMTS: A disintegrin and metalloproteinase with thrombospondin motif
Akt: Protein kinase B
ALP: Alkaline phosphatase
AMPK: Adenosine 5′-monophosphate-activated protein kinase
ARE: Antioxidant responsive element
BSP: Bone sialoprotein
C/EBP*α*: CCAAT/enhancer binding protein alpha
Col-I: Type I collagen
COX-2: Cyclooxygenase-2
CTGF: Connective tissue growth factor
EPCs: Endothelial progenitor cells
EPO: Erythropoietin
ERK: Extracellular regulated protein kinases
GLUT: Glucose transport protein
HFpEF: Heart failure with preserved ejection fraction
HIF-1*α*: Hypoxia-inducible factor 1alpha
hTRPC3: Human transient receptor potential cation channel subfamily C member 3
ICAM-1: Intercellular adhesion molecule-1
IFN-*β*: Interferon beta
IL-1*α*: Interleukin 1 alpha
iNOS: Inducible nitric oxide
I*κ*B*α*: NFKB inhibitor alpha
LPS: Lipopolysaccharide
MAO-B: Monoamine oxidase B
MCP-1: Monocyte chemoattractant protein-1
Medium MDI: Medium with M-CSF/dexamethasone/IL-4
MMPs: Matrix metalloproteinases
MnSOD: Manganese-superoxide dismutase
MRC1: Mannose receptor C-type 1
mPGES-1: Prostaglandin E synthase-1
MPTP: 1-Methyl-4-phenyl-1,2,3,6-tetrahydropyridine
MyD88: MYD88 innate immune signal transduction adaptor
NADPH: Nicotinamide adenine dinucleotide phosphate
NFATc1: Nuclear factor of activated T cells 1
NF-*κ*B: Nuclear factor kappa-light-chain-enhancer of activated B cells
NLRP3: NOD-, LRR-, and pyrin domain-containing protein 3
NO: Nitric oxide
Nrf2: Nuclear factor erythroid 2-related factor 2
OCN: Osteocalcin
PD: Parkinson's disease
PGE_2_: Prostaglandin E2
PKM2: Pyruvate kinase M2
PPAR*γ*: Peroxisome proliferator-activated receptor gamma
RANKL: Receptor activator of nuclear factor *κ*B ligand
ROR*α*1: Retinoid-related orphan receptor alpha1
STAT3: Signal transducer and activator of transcription 3
TGF-*β*: Transforming growth factor beta
TLR4: Toll-like receptor 4
TNF-*α*: Tumor necrosis factor alpha
Trx1: Thioredoxin
TXNIP: Thioredoxin interacting protein
VEGF: Vascular endothelial growth factor
VHL: von Hippel-Lindau.
